# Infectious Diseases and Vaccine Sciences: Strategic Directions

**DOI:** 10.3329/jhpn.v26i3.1897

**Published:** 2008-09

**Authors:** Stephen P. Luby, W. Abdullah Brooks, K. Zaman, Shahed Hossain, Tahmeed Ahmed

**Affiliations:** ICDDR, B, Mohakhali, Dhaka 1212, Bangladesh

**Keywords:** Communicable diseases, Diarrhoea, Mortality, Pneumoniea, Tuberculosis, Vaccine Aciences, Bangladesh

## Abstract

Despite substantial progress, infectious diseases remain important causes of ill-health and premature deaths in Bangladesh. Bangladesh has experienced a >90% reduction in the incidence of deaths due to childhood diarrhoea over the last 25 years. Further reductions can be achieved through the introduction of effective vaccines against rotavirus and improvements in home hygiene, quality of drinking-water, and clinical case management, including appropriate use of oral rehydration solution and zinc. Pneumonia is now the leading cause of childhood deaths in Bangladesh, and the pneumonia-specific child mortality is largely unchanged over the last 25 years. Reductions in mortality due to pneumonia can be achieved through the introduction of protein conjugate vaccines against *Haemophilus influenza* type b and *Streptococcus pneumoniae*, improvements in case management, including efforts to prevent delays in providing appropriate treatment, and the wider use of zinc. Tuberculosis is responsible for an estimated 70,000 deaths each year in Bangladesh. Although services for directly-observed therapy have expanded markedly, improved case finding and involvement of private practitioners will be important to reduce the burden of disease.

## INTRODUCTION

Vaccine-preventable diseases and other infectious diseases have historically been major causes of ill-health and premature deaths in Bangladesh. Over the last several decades, Bangladesh has made remarkable progress in reducing the burden of infectious diseases on human health, especially in children. Nation-wide infant mortality has decreased from 125 deaths per 1,000 births in 1978 to 65 deaths per 1,000 births in 1999–2003 (1,2). The rate of mortality among children aged less than five years (under-five mortality) in Matlab during 1975–1977 was 280 deaths per 1,000 births (3). This contrasts with a national under-five mortality rate of 88 in 1999–2003 (2) (Fig.).

**Fig.**
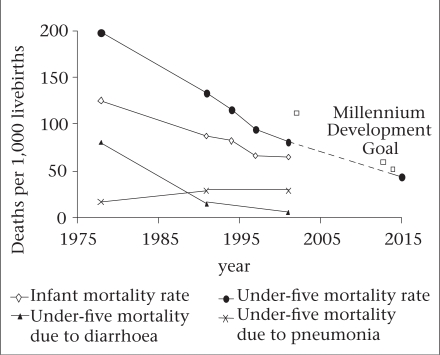
Under-five mortality rates in Bangladesh

These marked reductions in childhood mortality over the last 30 years are largely due to reduction in mortality from infectious diseases. The four interventions that appear to be most responsible for the decline in mortality include maternal tetanus toxoid vaccination (3-6), measles vaccination (7,8), and a reduction in diarrhoea fatalities due to oral rehydration solution (ORS) (9) and vitamin A capsules (10,11).

**Major points**

- Overall, rate of infectious diseases, as a cause of mortality, has declined substantially since 1990.
- Mortality due to diarrhoeal disease has declined by 90+% since 1975, but still needs to decrease further.
- Mortality due to pneumonia has not improved substantially and needs zinc and vaccine programmes to bring these rates down.
- Tuberculosis needs to be controlled before HIV/AIDS becomes prevalent.

Millennium Development Goal 4 (MDG 4) targets a reduction in under-five mortality by two-thirds between 1990 and 2015. The Bangladesh Demographic and Health Survey, covering the 1989–1993 period, measured under-five mortality at 133 deaths per 1,000 livebirths (12). If Bangladesh is going to meet Goal 4 of the MDGs, under-five mortality needs to be reduced to less than 50 per 1,000 livebirths by 2015^1^* (Fig.).

Despite substantial progress, infectious diseases remain important causes of ill-health and premature deaths in Bangladesh. In the Bangladesh Demographic and Health Survey 2004, 62% of deaths among under-five children in Bangladesh were ascribed to infectious diseases (Table 1) (2). This accounts for 55 deaths per 1,000 livebirths. If we expect a reduction in communicable diseases to account for its proportionate share of improvement in child mortality, childhood mortality due to infectious diseases needs to be reduced by 34 deaths per 1,000 livebirths, or a 38% reduction between 2000 and 2015.

**Table 1:** Causes of death among under-five children in Bangladesh

Cause of death	Proportion	
	1991–1993	1999–2003
Infectious		
Possible serious infection	10.1	31.2
ARI	14.9	21.1
Diarrhoea	8	5.1
Neonatal tetanus	6.6	2.3
ARI + diarrhoea	3.9	1.8
Measles	1.3	0.3
Measles with ARI or diarrhoea	3	0.3
Infectious subtotal	47.8	62.1
Non-infectious		
Birth asphyxia	22.5	11.7
Premature/LBW		6.5
Congenital abnormality		2.8
Birth injury		2.2
Injury	6.3	4.1
Malnutrition	6.6	3.6
Undetermined	16.8	2.3
Unspecified		3.2
Other causes		1.6
Non-infectious subtotal	52.1	38
Under-five mortality rate (deaths by age 5 years per 1,000 livebirths)	133	88
Death due to infectious diseases by age 5 years per 1,000 livebirths	63.6	54.6

ARI=Acute respiratory infection; LBW=Low birthweight

Infectious diseases are important beyond the age-group of children. MDG 6 has set a target that, by 2015, we will have halted and begun to reverse the incidence of malaria and other major diseases. Tuberculosis is a major cause of adult deaths, with an estimated 70,000 deaths per year in Bangladesh (13). Deaths of adults due to tuberculosis leave children orphaned and worsen the cycle of poverty and illness. There are other important multiple causes of morbidity and mortality due to infectious diseases in Bangladesh, including visceral leishmaniasis, malaria, Japanese encephalitis, and other meningoencephalitides. However, this strategic update will focus on three key areas that will require substantial progress to meet the MDGs; the key areas are: diarrhoeal diseases, pneumonia, and tuberculosis.

### Diarrhoea

#### Trend in burden of disease

- Mortality due to diarrhoea has declined by 90% since 1980, but is still too high.
- Bangladesh is a leader in ORS knowledge and practice.
- Use of zinc for diarrhoea needs to be scaled up rapidly.
- Rotavirus and cholera vaccines should be implemented as soon as possible and practical. A vaccine for enterotoxigenic *Escherichia coli* is needed.
- Cost-effective newer diarrhoeal treatments will become available soon—we should be ready to implement.
- Community-level hand-washing and water purification show great promise and need to be scaled up.

The earliest available population-based data on causes of deaths among under-five children in Bangladesh were collected from Matlab in 1975–1977. The diarrhoea-specific under-five mortality rate was 80 deaths per 1,000 children (46.6 due to watery diarrhoea and 33.4 due to dysentery) (3). Since methods for verbal autopsy have changed, we compared the current estimates by adjusting to make these similar to the previous estimates. Our adjustments for the current data assumed that all the deaths attributed to diarrhoea were, in fact, due to diarrhoea, that 50% of deaths attributed to diarrhoea combined with respiratory disease were due to diarrhoea, and that one-third of deaths due to combined measles with diarrhoea or respiratory disease were due to diarrhoea. With these assumptions, using the most recently-available national verbal autopsy data (1999–2003), diarrhoea accounted for 5.4 deaths per 1,000 children (12,14). Thus, Bangladesh has experienced a >90% reduction in the incidence of deaths due to childhood diarrhoea over the last 25 years.

This remarkable progress is likely due to a combination of factors, including improvements in nutrition, improved microbiological quality of drinking-water, following the substitution of surface-water sources for shallow tubewells, reductions in case-fatality ratio from the widespread use of oral rehydration solution (ORS), and wide-scale use of measles vaccine and vitamin A in primary healthcare programmes.

Nevertheless, 5.4 deaths per 1,000 births remain a substantial cause of childhood deaths in Bangladesh. Treatment of diarrhoeal disease consumes substantial resources, and the demand for ORS exceeds 200 million sachets annually. The increased prevalence of antimicrobial resistance, especially to *Shigella* species (15) and to *Vibrio cholerae* (16), threatens to complicate treatment and risks, increasing case-fatality ratios of patients who present for treatment.

#### Key issues contributing to burden of diarrhoeal disease

Several factors contribute to the ongoing severity of diarrhoeal disease in Bangladesh. First, the living environment, including foodstuffs and sources of drinking-water, is heavily contaminated with human enteric pathogens. The underlying forces that drive this contamination include poverty in the country and the high density of population. Bangladesh has the highest density of population compared to any country in the world that is not a small city state (17). Population growth and loss of land mass from global warming and elevated sea levels are expected to further markedly increase this density (see the paper on Population in this volume for additional discussion on the effects of global warming on Bangladesh).

Second, although there has been some improvement in the nutritional status of Bangladeshi children, malnutrition remains widespread, with 43% of children aged less than five years being stunted, that is below 2 standard deviations from the mean of the reference population, and 17% are severely stunted (18). Malnutrition increases the risk of diarrhoea and the risk of mortality from diarrhoeal disease (19-21).

A third factor that contributes importantly to the burden of diarrhoeal disease in Bangladesh is that the country provides a large natural ecological niche for *V. cholerae* and enterotoxigenic *E. coli* (ETEC)*.* Most vibrios are non-pathogenic, but some toxin-producing *V. cholerae* cause severe human disease. At the ICDDR, B hospital, *V. cholerae* causes 20-30% of cases of severe diarrhoeal disease each year (22). Since *V. cholerae* is endemic only in a few geographies globally, it has not attracted the same attention for vaccine development and prevention that other important pathogens have. ETEC is also common and occurs throughout the developing world, but is difficult for most laboratories to diagnose; so, it commonly goes unrecognized (23,24).

A fourth factor is the universal exposure to rotavirus, a serious pathogen that is disproportionately responsible for severe diarrhoeal illness in children aged less than two years. Recent analysis suggests that at least 38% of childhood deaths from diarrhoea are due to rotavirus (25). Surveillance from two typical upazila (subdistrict) health centres found that rotavirus was responsible for 13% of all hospital admissions of under-five children (Siddique K. Unpublished data). Unlike the bacterial pathogens, the burden of rotavirus will not lessen even if water and sanitation improves; it is common in developing and industrialized countries alike.

A fifth factor contributing to the burden of diarrhoeal disease is the lack of proper treatment for many children with severe diarrhoea. Although most people do have access to treatment facilities, many families choose to seek care from village doctors who do not use methods of modern treatment. For cholera, patients may die within a few hours, and there may not be time even to reach a facility before death.

#### Opportunities for reducing diarrhoeal mortality and morbidity

Reducing the incidence of childhood deaths from diarrhoeal disease should focus on three areas; (a) enteric vaccine development and introduction, (b) improvements in environmental health and hygiene, and (c) improved case management of diarrhoea. The three most important enteric vaccines for Bangladesh are vaccines against rotavirus, *V. cholerae*, and ETEC.

Two rotavirus vaccines have been proven to be safe and effective, preventing nearly all hospitalizations for rotavirus where they were tested in industrialized or middle-income countries. The vaccines have now been approved for use in the United States, Europe, and several other countries (26,27). ICDDR, B has found that the RotaRix rotavirus vaccine (from GlaxoSmithKline) is safe and immunogenic in Bangladesh, but further evaluations of its protective efficacy are needed to assist policy-makers in its introduction. The Centre is also evaluating the RotaTeq rotavirus vaccine (from Merck). Each of these studies with rotavirus vaccines is being conducted in collaboration with the Global Alliance for Vaccines and Immunization (GAVI) through its Rotavirus Vaccine Program and the World Health Organization (WHO). Other candidate vaccines, being developed in India and elsewhere, will likely be available within a few years, and their marketing may lower the price for effective vaccination.

Two major issues need to be addressed in order for these vaccines to substantially reduce mortality due to diarrhoeal disease in Bangladesh. First, the health benefit from the vaccines needs to be evaluated. The RotaTeq and RotaRix vaccines were effective in wealthier countries, but their effectiveness in poor countries with a large proportion of malnourished children, like Bangladesh, needs to be established. Factors, such as malnutrition, malabsorption, variation of serotypes, and the presence of other enteric agents might lessen the effectiveness of these vaccines. However, the vaccines could have a pronounced public-health benefit here by improving survival and growth of children and by lessening the need for health services.

Although rotavirus vaccines are estimated to be cost-effective in economic terms, these are still considered expensive compared to other vaccines covered by EPI. Thus, in addition to determining health outcomes, which are almost certain to be very positive, evaluation of rotavirus vaccines must include studies on the costing and financing mechanisms, so that the Government of Bangladesh can make a decision regarding their use.

Another type of vaccine appropriate for Bangladesh is a vaccine for cholera. ICDDR, B has been evaluating cholera vaccines for over 40 years, starting with injectable vaccines which had been used for nearly 100 years. Testing found these injectable vaccines to be impractical for public-health programmes. More recently, the Centre has been working with vaccines given orally, and two types are being evaluated. One is a live-attenuated vaccine called Peru-15 (28), and the other one is a killed oral vaccine similar to one licensed in Europe called Dukoral. Dukoral, tested in Matlab in 1985, was found to be safe and effective (29,30), but its high cost and logistic constraints have prevented its use in Bangladesh so far. Results from both killed and live vaccines are encouraging, and both may be useful in cholera-endemic areas such as Bangladesh. Although Peru-15 has the advantage that it can be used as a single dose, its storage, requires a cold-chain. Scientists at the Centre hope to evaluate this vaccine for efficacy within the next two years. With regard to the killed vaccine, the efforts now are to change the formulation to make it cheaper and easier to distribute. Although this killed vaccine will require two doses, it does not need to be kept cold, and it may even be suitable for ‘over-the-counter’ distribution.

ETEC also causes substantial rates of severe diarrhoeal disease (23,24). An effective vaccine would be expected to reduce mortality due to childhood diseases, but the current stage of research suggests that such a vaccine is unlikely to be available soon, although considerable effort is being invested in an ETEC vaccine (31). The Bill and Melinda Gates Foundation has recently announced that it will fund development of a vaccine for ETEC, and this funding will hopefully speed up its development.

The second intervention which is effective in reducing diarrhoeal disease relates to improving hygiene and sanitation. Although many feel that improved sanitation implies huge infrastructural costs, and in the long run, it will. In the short-term, however, much can be accomplished in the homes and villages. ICDDR, B performed much of the pioneering work on the effectiveness of intensive handwashing promotion on the reduction of diarrhoeal disease (32-34). A recent meta-analysis suggests that improved handwashing reduces the incidence of diarrhoea, on average, by 47% (35). However, approaches that have been quite successful in promoting improved handwashing on a small scale have not yet been demonstrated to be effective at a large scale.

Similarly, efforts to improve the quality of drinking-water through point-of-use purification can markedly reduce diarrhoea (36). Again, the approaches need to be demonstrated to be effective at a larger scale (37). Both poverty and the density of population in Bangladesh make effective environmental sanitation difficult. Various approaches are being used in Bangladesh; however, there has been little critical evaluation of their effectiveness in reducing diarrhoeal disease. Most important will be efforts to change community norms through explaining how to effectively reduce contaminations and how to purify and store water. Thus, efforts to creatively deliver and assess behaviour-change messages at scale will be critical.

The third intervention to reduce diarrhoeal mortality is improved case management of patients with diarrhoea. This encompasses improving strategies to increase recognition of serious diarrhoea by mothers with appropriate home-treatment, and developing and diffusing appropriate clinical management of diarrhoea, including the appropriate use of ORS and zinc throughout the country. Bangladesh benefits from a strongly-held view of the benefits of ORS, while its use has been slipping in many other countries. However, the use of unnecessary antibiotics is still common. As the use of zinc increases, it is hoped that the use of unnecessary antibiotics will decrease. In addition, newer therapies, including some based on traditional remedies, and including amylase-resistant starches (using green bananas as a readily-available source of amylase-resistant starch) appear to effectively reduce the duration of diarrhoea and prevent the development of persistent diarrhoea (38). The introduction of zinc will be critical, and the other remedies will also be useful as these are validated and products made available.

### Pneumonia

#### Trends in burden of disease

Pneumonia is the leading cause of childhood deaths globally, accounting for two million deaths per year among children aged less than five years in 2000–2003 (39). In Matlab, the pneumonia-specific under-five mortality rate, during 1975–1977, was 16.8 deaths per 1,000 children (3). In the most recent data (1999–2003), pneumonia accounted for 28.6 deaths per 1,000 children.† Thus, in striking contrast to a marked reduction in diarrhoea-specific mortality among Bangladeshi children, the incidence of pneumonia-specific child mortality is largely unchanged over the last 25 years (Fig.). Respiratory disease now accounts for five times as many deaths among Bangladeshi children as diarrhoeal disease. If Bangladesh is going to meet the MDG set for under-five mortality by 2015, prevention of deaths due to pneumonia must be targeted.

Both bacteria and viruses are aetiologic agents responsible for pneumonia. The most common viruses include influenza A and B, respiratory syncytial virus (RSV), and metapneumovirus (40-42). The bacterial causes are *Streptococcus pneumoniae* and *Haemophilus influenzae* type b (Hib). Although there is not a complete understanding of the agents responsible for pneumonia in each setting (urban vs rural and age-specific rates), the picture is becoming more clear. Viruses are detected more commonly, but the bacteria tend to cause more severe disease by the time they are detected (43). However, since viral infections are so much more common (by a factor of 23 when comparing influenza and *S. pneumoniae* during our urban surveillance), viruses are, in fact, estimated to be the most common cause of severe pneumonia (44,45). Influenza virus is especially associated with severe illness and hospitalizations of young children (45-48). Additionally, evidence from both human and animal studies have shown that pneumococcal infection is more severe, even lethal, following an influenza infection, suggesting the need to better understand the possible interactions of the two types of agents (49,50).

- Pneumonia has overtaken diarrhoea as the number 1 infectious killer of children.
- Case management needs to improve to prevent delays within the family and by providers.
- Zinc treatment for diarrhoea will prevent deaths from pneumonia.
- Vaccines for *H. influenzae* and S. *pneumoniae,* and possibly influenza virus will save lives.
- The impact of the introduction of the new pneumonia vaccines needs to be monitored.

Although paediatric pneumonia is the main issue with regard to MDG 4, pneumonia is also a major health problem for the elderly (51); however, this has not been carefully studied in developing countries. Data from developed countries suggest that pneumonia is common, indicating that it is likely a substantial cause of disease among adults in Bangladesh as well.

#### Key issues contributing to burden of pneumonia

Despite considerable progress in understanding the aetiologies and clinical management of individual patients with pneumonia, this understanding has not yet resulted in a reduction in pneumonia-specific child mortality. The cornerstone of the public-health response to childhood respiratory disease has been attempting to scale up clinical case management for children with signs of pneumonia. For individual patients, appropriate case management is critical, but, as illustrated by the minimal improvement in the burden of overall disease, this approach has not been effective in Bangladesh in lowering deaths due to pneumonia. Whether this limited benefit is due to lack of seeking healthcare, lack of access to treatment when needed, or due to ineffectiveness of the treatment provided is not that clear. Effective treatment is complicated by the widespread emergence of resistance to first-line antimicrobial agents (52,53), but it is also clear that parents recognize the signs of severe respiratory infection at a slow pace; so, proper treatment cannot be sought earlier in the course of the disease.

Two factors contribute to the increased prevalence of antimicrobial resistance (54,55): (a) high rates of antimicrobial exposure among young children due to pneumonia and febrile illnesses and (b) inappropriate (unnecessary) and inadequate (dosing) antimicrobial exposure. One factor that could reduce inadequate exposure and enhance compliance would be a shorter course of treatment of pneumonia, which is under consideration (56). Another that could assist in reducing multiple antibiotic exposures per illness would be to reduce treatment failure. New adjuvant therapies with zinc that have shown promise in this regard (57) and in shortening the duration of illness should be further studied.

A barrier to reducing mortality due to pneumonia is the inherent difficulty in the recognition of disease, especially in recognizing danger signs. An infant with pneumonia often does not appear seriously ill to parents until it is too late for the child to receive life-saving medical therapy. In contrast to diarrhoea, in which the symptoms and signs are obvious to parents, the signs of pneumonia may not be recognized. When they are recognized, caregivers may not see them as abnormal, or they may delay effective treatment by changing diets first, or seeking help from a spiritual healer. These were barriers to effective treatment for diarrhoea many years ago, but knowledge regarding dehydration and the effectiveness of ORS is now nearly universal in Bangladesh. Similar knowledge regarding respiratory infections is needed so that the danger signs can be recognized earlier and care sought.

#### Opportunities for major impact on mortality due to pneumonia

Mortality due to pneumonia can be reduced substantially if new vaccines and improved case-management strategies could be employed. The potentially vaccine-preventable illnesses include those due to *H. influenzae*, *S. pneumoniae*, and influenza. Additionally, mortality due to pneumonia can be reduced substantially by using zinc properly. The vaccine for *H. influenzae* is the protein-conjugate Hib (58,59) and, for *S. pneumoniae*, is a similar conjugate pneumococcal vaccine (60,61). A Hib vaccine-probe study in Bangladesh demonstrated that the Hib vaccine reduced the incidence of pneumonia by 30% and meningitis by 50% (62). Another trial of the Hib conjugate vaccine in the Gambia reduced severe pneumonia by 21% (63). A conjugate pneumococcal vaccine reduced the incidence of pneumonia by 37% and reduced documented pneumococcal infections (all serotypes) by 50%. Overall mortality was reduced by 16% in the Gambian study. The conjugate pneumococcal vaccine has not been evaluated in settings where pneumonia is a major cause of child mortality in Asia; however, the ICDDR, B study area in Dhaka is making preparations for this kind of study.

Regular administration of zinc provides an aetiology-independent intervention to prevent severe pneumonia, and two different strategies have both shown effectiveness. In one ICDDR, B study, a daily dose of zinc was prescribed for 14 days for each episode of diarrhoea. Since many children have repeated bouts of diarrhoea, many children in the intervention villages received several courses of zinc. Follow-up of these children in the intervention villages, along with a similar group in the control villages, revealed that rates of future episodes of pneumonia were reduced, and importantly, the overall rate of mortality was reduced by about 50% (64). A second ICDDR, B study in urban Dhaka administered a weekly dose of zinc in a double-blind, placebo-controlled, individually-randomized trial to determine if this would reduce rates of diarrhoea and pneumonia. This strategy resulted in a remarkable >80% reduction in overall mortality (65). The reduction in deaths was explained by a reduction in pneumonia. Since zinc gives aetiology-independent protection, one does not have to adjust for species or serotypes of the pathogen; however, the strategy for administration of zinc needs to be consistent. While vaccines can be given in two or three doses, providing prolonged protection, the zinc therapy needs repeated dosing and consistent application. We believe that this is possible with adequate marketing and education (66,67).

These new strategies—the new conjugate vaccines and zinc—hold substantial promise to prevent mortality due to pneumonia, but these have not yet been scaled up. ICDDR, B is involved in scaling up zinc therapy for diarrhoea, and we will continue to follow its impact on prevention of severe pneumonia. We anticipate that the Hib vaccine will soon be introduced in Bangladesh, and the Centre has received funding to monitor the vaccines impact. The pneumococcal vaccine is also available at below market prices, and we look to its introduction in Bangladesh. The Centre is well-placed to assess the impact of the introduction of conjugate pneumococcal vaccine and to participate in the evaluation of future pneumococcal vaccines that would protect even more children than the currently-available vaccines.

Improvements in case management are also possible both from the standpoint of improving quali-ty of treatment and making this treatment available to children who need it. Since most children who die of pneumonia do not receive standard care, they die at home or are brought to treatment facilities too late. Why is this happening? Formative research is needed to understand perceptions of respiratory illness and to help understand how to communicate danger signs of pneumonia. Since the symptoms may not be so obvious, or may be perceived to have other causes, clear health messages need to be developed which will allow parents to recognize pneumonia, to help them decide to seek care from an adequately-trained health provider, and then to take action to avail of this care. Our community-based studies found that most children with pneumonia, who are referred to the hospital or clinic by the health worker, do not actually visit them. Reasons for this behaviour must be understood to address the constraints to accessing proper treatment.

Considering the huge number of children with pneumonia, sufficient facilities are not available for treating them if all who were supposed to be hospitalized actually visited. Thus, alternative treatment strategies are needed to handle the case load. ICDDR, B has been evaluating a day-care model for patients with severe pneumonia who are refused admission in the hospital for lack of space (ICDDR, B. Unpublished data). The information to date suggests that this method is as effective as hospital care. Rarely, the condition of a patient will deteriorate, requiring admission, but more than 98% are successfully treated in the day-care facility. Although formal costing is needed, treatment in such a facility is likely to be much more cost-effective and acceptable when compared with a hospital.

#### Moving forward

With the availability of new vaccines, the strategies with zinc, and more intensive treatment programmes, it should be possible to significantly reduce pneumonia by at least 50% in a short period. Among the interventions listed, scaling up zinc is currently feasible and is in the process of implementation. The routine use of Hib vaccine will soon commence in Bangladesh, and the success of this intervention needs to be closely monitored. During the introduction phase, we anticipate that GAVI funding will pay for the costs, but impact data are needed to justify subsequent continued national expenditure. We look for the pneumococcal vaccine to be introduced with GAVI support within 12-24 months after the introduction of Hib vaccine, and again careful assessment of impact will be crucial to support continued vaccination.

Influenza vaccine is not generally being considered for use among children in a developing country, but our data suggest that this may be a powerful intervention if it could be introduced. Since influenza-associated infections likely predispose to bacterial infections, it is possible that an influenza vaccine may provide broader-spectrum protection than simply against influenza. Results of preliminary studies in which the influenza vaccine was given to pregnant women suggested that children born to these mothers had fewer respiratory infections during the first six months, perhaps by virtue of placental or breastmilk antibodies from the mother (ICDDR, B. Unpublished data).

### Tuberculosis

- Bangladesh's burden of disease from tuberculosis ranks number 6 in the world.
- The DOTS programme with government and non-government partners provides services.
- Excellent support from WHO and GFATM for tuberculosis is available.
- Need for basic data on the prevalence, incidence, and risk factors to better plan control strategy.
- Need to include private providers as partners in the overall control strategy.
- Need for active case finding to increase proportion of cases detected.
- Paediatric tuberculosis is generally not being diagnosed or treated.

The WHO has declared tuberculosis as a global health emergency; the first infectious disease to be designated as such (68). Tuberculosis remains a leading cause of morbidity and mortality in developing countries, including Bangladesh. About 7% of all deaths in developing countries are attributed to tuberculosis, and it is the most common cause of death from a single source of infection among adults (69). In a recent analysis of the global burden of tuberculosis, Bangladesh ranked the sixth highest among 212 countries in 2004 (13) having 300,000 new cases and around 70,000 deaths annually. However, there are limited systematically-collected epidemiologic data from Bangladesh. While directly-observed treatment, short-course (DOTS) is provided by the Government, often with assistance from NGOs, effective control of the burden of disease is hampered by the fact that only 46% of cases are detected (70). The previous tuberculosis-prevalence survey conducted by the National Tuberculosis Control Programme during 1987–1988 estimated a prevalence of sputum-positive tuberculosis of 870/100,000 in the population of ≥15 years old. It was higher among men (1,008/100,000) than among women (600/100,000) and higher in urban areas (1,610/100,000) than in rural areas (800/100,000) (72). A recently-completed population-based surveillance in the Matlab field area of ICDDR, B found a much lower prevalence of tuberculosis, i.e. 95/100,000 among persons aged ≥15 years (76). The estimate for incidence from the WHO documents which showed an annual incidence of tuberculosis in Bangladesh was estimated to be 229 per 100,000 (13).

Tuberculosis occurs more frequently among low-income populations living in over-crowded, poor neighbourhoods, and people with little schooling (77). The available data on the impact of tuberculosis-control measures on the prevalence of the disease in Bangladesh is limited (78). With the impending threat of the human immunodeficiency virus (HIV) epidemic reaching Bangladesh (79), there is a risk for a substantial increase in the incidence of tuberculosis.

The prevalence of sputum-smear-positive tuberculosis estimated by various studies is shown in Table 2. The wide variations in the prevalence of tuberculosis are likely due to differences in survey methods, population, and age-groups studied and time periods.

**Table 2:** Prevalence of sputum-positive tuberculosis estimates in different surveys

Period	Organization	Prevalence estimates* and reference
1964–1966	Directorate General of Health Services, Bangladesh	318 (71)
1987–1988	Directorate General of Health Services, Bangladesh	870 (72)
1995	BRAC	70-150 (73)
2001	Damien Foundation	24 (74)
2001	ICDDR, B	95** (75)

*The estimate is per 100,000 people of ≥15 years of age, except for Damien Foundation where the corresponding age of people was ≥12 years

**Tuberculosis cases receiving treatment but not having a cough of >3 weeks at the time of the survey were not included. Therefore, some prevalent cases might have been missed. If these cases were included, the prevalence would be 111/100,000

There is a marked gender difference in tuberculosis, with men being much more commonly affected than women. Globally, the ratio of female-to-male tuberculosis cases notified is 0.47-0.67 (81). In 2004, countries reported 1.4 million smear-positive cases in men, but only 775,000 in women (82). The male predominance for AFB-positive sputum in Bangladesh is particularly pronounced (Table 3). Although men may be able to produce adequate sputum specimens more readily than women, and they may also be able to access treatment facilities more easily, there does appear to be a real difference in rates of the infection that is not understood. A relationship with other environmental hazards, e.g tobacco, has been established as one potential predisposing factor that might partially or largely explain the gender difference (83).

**Table 3:** Female-male ratio of sputum smear-positive cases in different studies conducted in Bangladesh

Study	Year of study	Sputum AFB-positive female/male ratio and reference no.
BRAC	1992–1994	0.39 (73)
NTP	1997	0.35 (80)
Damien Foundation	2001	0.33 (74)
ICDDR, B	2001	0.24 (75)

AFB=Acid-fast bacilli

Tuberculosis affects both adults and children, but its diagnosis is particularly difficult in children in whom the signs and symptoms seem to be non-specific. This makes the assessment of the magnitude of childhood tuberculosis uncertain. An estimated 88 million cases of tuberculosis occurred in the past decade globally; of them, 15 million were children, and five million of them died (84). Data on tuberculosis among children in Bangladesh are limited. Of the total number of newly-detected sputum-smear-positive cases reported in 2000, children aged less than 14 years constituted only 2%. This is a gross under-estimate because young children cannot effectively produce sputum, and sometimes their infection is extrapulmonary. A recent review of the National Tuberculosis Control Programme of Bangladesh has, therefore, recommended research on childhood tuberculosis—its diagnosis and association with malnutrition—as a priority (85).

Children acquire tuberculosis from droplet infection with *Mycobacterium tuberculosis* spread from aerosol formation of sputum expectorated by adults with pulmonary tuberculosis. A smear-positive (presence of acid-fast bacilli, *M. tuberculosis*, in the sputum as detected by microscopy) pulmonary tuberculosis patient may infect 50-70% of children aged four years or less. However, following infection, not all children develop the disease, characterized by clinical or radiological abnormalities. Approximately 10-30% of the infected children develop the disease in the 3-12 months following infection (86). The risk of the disease is increased by factors, including younger age of the child, malnutrition, second-hand smoke or indoor air pollution, or any condition that compromises immune function, i.e. measles.

### Key issues contributing to burden of tuberculosis

#### Inaccurate assessment

As noted above, assessments of the prevalence of tuberculosis vary widely in Bangladesh. Without a sound assessment of its burden, it is difficult to assign appropriate priority to the problem and to assess the effectiveness of interventions. For example, case detection for tuberculosis is calculated dividing the number of persons who are treated in the National Tuberculosis Control Programme by the estimated prevalence of smear-positive tuberculosis in the country. Detection of cases has increased from 29.2% in 1993 to 46% in 2004 (70). Clearly, more cases are being detected, although detection remains below the target of 70%. However, the uncertainty of the underlying estimate of tuberculosis means that the case-detection rate could be at the programme target or very far below. The absence of data undercuts sound allocation of resources within the National Tuberculosis Control programme. A nationwide tuberculosis-prevalence survey is being undertaken by WHO and ICDDR, B which will provide better estimates of the burden of the disease and from the basis for strengthening tuberculosis-control strategies in the country.

The prevalence of tuberculosis is not the only key epidemiologic data that are lacking, we also do not understand the pattern of transmission in Bangladesh. For example, we do not understand why males have so much more tuberculosis than females or the settings where tuberculosis is most efficiently transmitted. Innovative efforts to reduce tuberculosis require better understanding of its epidemiology. The male predominance suggests certain risk factors or transmission mechanisms that, if understood, could provide intervention strategies. The rapid urbanization with its increasing crowding will also be critical to understanding the patterns of tuberculosis spread.

#### Emerging drug resistance

The majority of patients who are enrolled in the National Tuberculosis Control Programme have a good clinical outcome. Of patients identified in 2003, 83% were cured, 5% died, 5% defaulted, and 1% failed therapy.^$^ The few studies that have been conducted in Bangladesh have identified relatively low levels of drug-resistant *M. tuberculosis*. The prevalence of resistance of *M. tuberculosis* to any anti-tuberculosis drug has ranged from 18.6% to 48.4%. Multidrug-resistant tuberculosis (MDR-TB) defined as resistance to both isoniazid and rifampicin ranged from 2.0% to 5.5% (Table 4) (76). In addition, recent molecular typing of *M. tuberculosis* strains has identified Beijing family isolates (89). These isolates are highly drug-resistant and appear to represent more recent transmission within Dhaka. MDR-TB is associated with a death rate of 50-80% and often with a short disease span (4-16 weeks) from diagnosis to death. Treatment of these cases is also much more expensive—estimated to be from US$ 800 to $10,000 in a developing country. With an estimated 10,000 MDR-TB cases in Bangladesh, this will be a substantial burden to control.

**Table 4:** Drug-resistance patterns of *Mycobacterial tuberculosis in* different studies conducted in Bangladesh

Study	Resistance to any drug (%)	Rates (%) of multiple drug resistance and reference
BSMMU	29.7	4.9 (87)
Damien Foundation	18.6	2 (88)
ICDDR, B	48.4	5.5 (76)

BSMMU=Bangabandhu Sheikh Mujib Medical University; ICDDR, B=International Centre for Diarrhoeal Disease Research, Bangladesh

#### Difficulties in identifying cases

From a programmatic sense, the detection of tuberculosis cases is difficult. Persons should be screened if they have a persistent cough for more than three weeks, and a sputum sample should be tested under a microscope using acid-fast staining methods.** Although this system should be detecting especially those patients who are most likely to transmit the infection, there are several constraints which limit its success as a public-health strategy in Bangladesh. The first delay in case identification is the delay in the patient or family recognizing that these symptoms require investigation. The second delay is in finding a clinic or a provider which can deal with the symptoms appropriately. The third delay is the competence of the laboratory in accurately carrying out the test. The fourth delay may occur if, after diagnosis, the patient is not enrolled in a properly-organized DOTS programme to supervise the treatment during the treatment period. If the system is working properly, it will identify persons who are most likely to transmit infection, but it also misses many persons who will go on to develop more severe disease and continue to transmit their infection to others.

#### Tuberculosis in children

The DOTS strategy should reduce the burden of the disease in adults, but it does not target children. As a result, there is a lack of consistent programmatic strategy for children, and childhood tuberculosis remains an under-diagnosed and neglected entity. In developing countries, most cases of childhood tuberculosis are associated with malnutrition, poverty, and ignorance. Poverty not only leads to malnutrition but also to over-crowded housing, poor ventilation, indoor air pollution, and poor hygiene habits, such as coughing and spitting sputum in the open.

The diagnosis of tuberculosis in childhood is particularly difficult because clinical manifestations of symptoms in children are usually non-specific. Unlike adults, infants and young children usually do not develop the cavitary form of pulmonary tuberculosis which is easily detected by a chest X-ray and from which *M. tuberculosis* enter the bronchi to be expectorated in the sputum. Moreover, young children cannot produce sputum. Malnutrition, which is so common in Bangladesh, not only predisposes children to tuberculosis and makes it more severe, but also further confounds the diagnosis of tuberculosis because the tuberculin skin test for tuberculosis (PPD) often gives a false-negative result in malnourished patients (90). The skin test is also less useful because of the BCG immunization that is universally given in Bangladesh. Even immunocompetent and well-nourished children with confirmed tuberculosis occasionally (10%) may have a negative skin test (91).

There are clinical scoring systems for the diagnosis of childhood tuberculosis (92), but these are little used by general practitioners who see most sick children in Bangladesh. The sensitivity and specificity of the scoring systems are difficult to assess.

#### Role of the private sector

More than 80% of clinical care in suspected tuberculosis cases in Bangladesh is provided by the private sector. Many general practitioners have limited knowledge of the necessary steps to diagnose and treat tuberculosis. Anti-tuberculous medications are available over-the-counter throughout Bangladesh. Patients who are misdiagnosed continue to spread the disease to their contacts, and patients who are incompletely treated are at a high risk of developing drug resistance. Unfortunately, only a few of those presenting to private care providers, including non-graduate (non-licensed) private practitioners, pharmacists, indigenous practitioners, and graduate private practitioners) were properly evaluated and referred for a DOTS facility (ICDDR, B. Unpublished data).

#### Moving forward

Bangladesh is at a crucial stage in the epidemic of tuberculosis. Considerable resources are becoming available through the National Tuberculosis Control Programme, and there is a good partnership with NGOs, including BRAC and the Damien Foundation and other NGOs, including the research and quality control provided through ICDDR, B. The entire effort is being assisted with the Global Fund to AIDS, Tuberculosis and Malaria. The cooperative efforts between these groups are an encouraging sign, and we hope that this will bring results that have not been seen previously. This cooperative effort will give the DOTS strategy a major boost.

Several critical research questions, if properly engaged and answered, can translate into saving of hundreds of thousands of lives.

#### Improved assessment

ICDDR, B is collaborating with the Government of Bangladesh and WHO to update the national estimate of the prevalence of tuberculosis. This is a first step towards improving the understanding of the tuberculosis problem in Bangladesh. The Centre's experience in population-based surveillance, its surveillance sites at locations throughout the country, its laboratories which have techniques for molecular typing, and a BSL-3 environment to safely handle specimens provide an excellent platform to study key issues in the transmission of the disease.

These studies will allow for a better understanding of the pathways of transmission, elucidating why men have so much more tuberculosis than women and developing new ways to identify people who are particularly efficient at spreading tuberculosis. It was clear from GIS data from Matlab that the prevalence of tuberculosis tended to occur in clusters, and understanding this clustering would help in case-finding in the future.

#### Emerging drug resistance

Monitoring drug resistance will be critical to ensure that resistant strains are not allowed to spread. Such strains are now occurring in Bangladesh, but are not yet a major problem, except in ‘retreated’ cases. Although the WHO provides guidelines for the treatment of resistant cases, there will be a continued need to monitor the practice of the system to insure that the guidelines are being followed. Further, laboratory testing is needed to continually update information on resistance patterns so that rationale drug treatments can be used. This information will be provided to the National Tuberculosis Control Programme and WHO.

#### Accelerated identification of tuberculosis cases

Although Bangladesh has been quite effective in treating cases once detected, many persons who are infected are not being detected. According to the national estimates, the case-detection rates have been improving nationally; however, there is still much room for more improvement. Identifying cases earlier in their illness will require a combination of approaches that address the ‘delays’ mentioned earlier. Programmes can improve communications to alert people who have persistent cough to seek a test. These patients must have ready access to the tests, and the tests performed must be reliable. Finally, once a patient with tuberculosis is identified, he/she must be able to quickly enroll in an approved DOTS programme. Each of these steps must be improved but each will require concerted efforts by more fully understanding the delays that have existed. It would seem that media communications are needed for the first and the second delay, but improved health services and quality-control methods are needed for the last two. Rather than assuming that these interventions are appropriate, operations research is, however, needed to validate the causes and solutions for the delays.

One approach which is not currently employed widely in Bangladesh is contact-tracing of tuberculosis cases. In many other countries, family members and other close contacts of active cases are screened to determine if the infection had been spread to these other members. This practice is not commonly done in Bangladesh, partly because of logistic/manpower constraints and partly because of the lack of suitable screening methods. At this point, only symptomatic screening can be done, sometimes enhanced by skin testing. We know, however, that these contacts could be harbouring the infection without showing signs of the disease. Only more sophisticated tests could detect these incubating infections.

#### Improved diagnostics

Improved diagnostic tests, especially tests that could identify patients with active disease before they begin transmitting the organism to others, could revolutionize the control of tuberculosis. Scientists at ICDDR, B have developed a new technique that uses cultures of peripheral blood mononuclear cells and detects tuberculosis-specific antibodies in lymphocyte supernatant (ALS) for the diagnosis of active pulmonary tuberculosis in adults (93). If this approach could be developed into a low-cost rapid test, it could markedly improve the clinical management of tuberculosis.

Working with FIND, ^$$^ ICDDR, B is validating a rapid and practical DNA-based test to diagnose tuberculosis from sputum. This test could also speed the diagnosis.

#### Tuberculosis in children

Diagnosis of tuberculosis in children remains an enigma. The laboratory test mentioned above could considerably simplify this effort. Pending the development of such tests, validation of the scoring system may offer the best opportunity to identify cases early (91). Indeed, reducing childhood tuberculosis may contribute importantly to meeting MDG 4.

#### Health systems research for especially vulnerable groups

Tuberculosis is not randomly distributed, and efforts are needed to devote resources to those who are most vulnerable, including interventions to extend DOTS to hard-to-reach areas; strengthening urban DOTS; improving accessibility to DOTS by high-risk populations (in prison, factory workers, persons with HIV/AIDS, the extremely poor); and optimizing the role of private practitioners in the control of tuberculosis.

#### New vaccines

Although new vaccines are not yet available, Bangladesh will be a logical site to test such vaccines when they do become available. For the short-run, the control programmes will, however, depend on the detection of cases, assuring that these patients receive adequate treatment through DOTS.

## Footnotes

* The official MDG 4 set by the Government of Bangladesh is 50% according to the Poverty Reduction Strategy Paper. Depending on the survey used for establishing the ‘baseline’ in 1990, the target could be slightly more or less, but 50% is the official target

† Since the method for classifying deaths using verbal autopsies has changed over the years, we made adjustments to make the different years comparable. To do this, we assumed that all deaths attributed to respiratory infection were due to pneumonia, that 50% of deaths attributed to diarrhoea combined with respiratory disease were due to pneumonia, that one-third of deaths due to combined measles with diarrhoea or respiratory disease were due to pneumonia, and that one-third of deaths from possible serious infection were due to pneumonia

^$^ A patient is considered to have failed if the signs and symptoms persist and if the sputum smear continues to be positive for five months or later after starting treatment

** Other common methods for the diagnosis of tuberculosis include a clinical diagnosis based on signs and symptoms combined with a chest X-ray. Sputum culture is possible in Bangladesh, but is not readily available. Culture methods increase sensitivity but results are not available for 4-6 weeks

^$$^ FIND is a WHO-affiliated public-private partnership based in Geneva which is developing new diagnostic tests for tuberculosis
